# Synovial fluid o-tyrosine is a potential biomarker for autoimmune-driven rheumatoid arthritis

**DOI:** 10.1007/s10067-025-07491-z

**Published:** 2025-05-31

**Authors:** Anne-Mari Mustonen, Juha Savinainen, Marko Lehtonen, Petri Lehenkari, Tommi Kääriäinen, Antti Joukainen, Heikki Kröger, Petteri Nieminen

**Affiliations:** 1 Institute of Biomedicine, School of Medicine, Faculty of Health Sciences, University of Eastern Finland, Kuopio, Finland; 2 Department of Environmental and Biological Sciences, Faculty of Science, Forestry and Technology, University of Eastern Finland, Joensuu, Finland; 3 School of Pharmacy, Faculty of Health Sciences, University of Eastern Finland, Kuopio, Finland; 4 Translational Medicine Research Unit, Faculty of Medicine, University of Oulu, Oulu, Finland; 5 Medical Research Center, University of Oulu and Oulu University Hospital, Oulu, Finland; 6 Department of Surgery, Oulu University Hospital, OYS, Oulu, Finland; 7 Pihlajalinna, Kuopio Finland; 8 Department of Orthopaedics, Traumatology and Hand Surgery, Kuopio University Hospital, Kuopio, Finland; 9 Kuopio Musculoskeletal Research Unit, University of Eastern Finland, Kuopio, Finland

**Keywords:** Amino acid, Infrapatellar fat pad, Osteoarthritis, Rheumatoid arthritis, Synovial fluid

## Abstract

**Introduction/objectives:**

The aim of the present study was to identify key amino acid (AA) pathways in osteoarthritis (OA) and autoimmune-driven rheumatoid arthritis (RA), as AAs have emerged as potential biomarkers for the detection of degenerative joint diseases. It was hypothesized that we would detect distinct metabolic pathways activated in OA and RA due to different degrees of inflammation.

**Method:**

Samples of synovial fluid (SF) and infrapatellar Hoffaʼs fat pad (IFP) were collected from end-stage knee OA (*n* = 10) and RA patients (*n* = 10), and from non-inflammatory controls (*n* = 5). Metabolites were analyzed utilizing a liquid chromatography high-resolution mass spectrometry approach, followed by univariate and multivariate statistical testing and pathway analysis by MetaboAnalyst. Receiver operating characteristic analysis was used to examine diagnostic values.

**Results:**

SF results identified o-tyrosine as a promising biomarker for distinguishing RA patients from OA patients and controls, and cystine, cysteine, and methionine separating OA patients from controls. Regarding IFPs, *β*-alanine could have diagnostic value to discriminate RA and OA. The present data indicate alterations in metabolic pathways, such as cysteine and methionine metabolism in RA and OA SFs compared to control SF, selenocompound metabolism in RA *vs.* OA SFs, and pyrimidine metabolism in RA *vs.* OA IFPs.

**Conclusions:**

The identified nitrogen compounds, particularly o-tyrosine, and AA metabolism pathways have potential as novel diagnostic and therapeutic targets for degenerative joint diseases.

**Table:** 

**Key Points** •* Synovial fluid o-tyrosine can distinguish rheumatoid arthritis from osteoarthritis and control.* • *Synovial fluid cystine, cysteine, and methionine separate osteoarthritis from control.* • *β-Alanine in intra-articular fat has diagnostic value between rheumatoid arthritis and osteoarthritis.* • *A routine measurement of o-tyrosine would be useful in the future as an indicator of rheumatoid arthritis.*

**Supplementary Information:**

The online version contains supplementary material available at 10.1007/s10067-025-07491-z.

## Introduction

Osteoarthritis (OA) and autoimmune-driven rheumatoid arthritis (RA) are joint diseases characterized by low- and high-grade systemic inflammation, respectively [1–3]. They share partly similar symptoms but have different, still inadequately understood pathogeneses [4, 5]. Adipose tissues, including the intra-articular and infrapatellar (Hoffaʼs) fat pad (IFP), might contribute to the progression of these chronic arthropathies [6]. IFP adipocytes and infiltrating immune cells may release inflammatory and degradative factors, such as adipocytokines and fatty acids, to synovial fluid (SF) that is in direct contact with the synovial membrane and articular cartilage. These agents have been suggested to influence the behaviour of joint tissues to stimulate the production of inflammatory factors and cartilage-degrading proteinases, and to decrease the synthesis of proteins constituting the cartilage extracellular matrix.

One fundamental challenge for arthritis research is to identify specific, sensitive, and reliable biomarkers to detect these diseases in their early stages. Metabolomic profiling is a clinically promising approach for this purpose, with potential applications in both diagnosis and therapy monitoring [7]. In addition to metabolites of carbohydrate and lipid metabolism, amino acids (AAs) have emerged as putative biomarkers for the detection of joint diseases [8], and there are OA-related alterations in the profiles and metabolism of, for instance, glutamic acid and arginine family AAs [9]. Overactivity of the arginine-to-ornithine pathway may lead to arginine depletion, resulting in an imbalance between cartilage repair and degradation [10]. Metabolic pathways of alanine and branched-chain AAs (BCAAs: valine, isoleucine, and leucine) are also altered in OA [10, 11]. Circulating BCAAs can increase in OA but decrease in RA with potential in the differential diagnosis of these conditions [11], and the ratios of BCAAs to histidine have been suggested as biomarkers for knee OA (KOA) [12]. Significant associations between systemic AA concentrations and articular cartilage thickness, KOA pain, physical performance, sensorimotor function, and mental health, independent of age and body adiposity, were recently documented [13]. Regarding IFP, pathways of AA and histamine metabolism were suggested to be downregulated in RA compared to KOA [7].

The aim of the present study was to examine the differences in local AA metabolism in SF and the adjacent IFP in KOA and RA. The obtained data could be a useful starting point to translational studies to assess potential biomarkers for early-stage diseases and to identify targets for therapeutic intervention. It was hypothesized that (*i*) based on the different degrees of inflammation, we would detect distinct metabolic pathways activated in KOA and RA, and that, in addition to diagnosis-related variation, (*ii*) there would be differences specific to IFP or SF regarding the potential biomarkers for these joint diseases.

## Methods

### Ethics, patients, and sampling

**Fig. 1:**
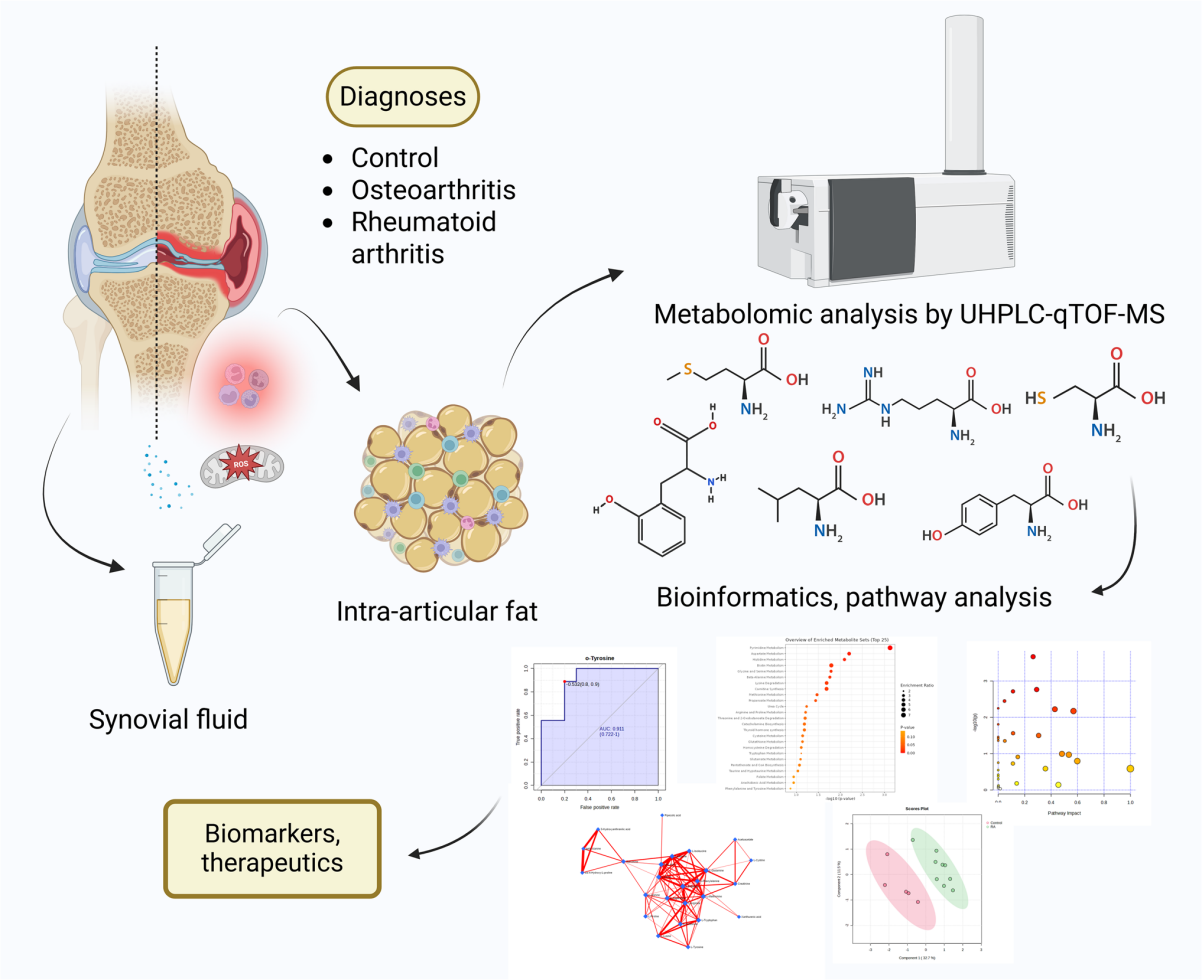
Illustration summarizing the study protocol. UHPLC-qTOF-MS = ultra-high-performance liquid chromatography quadrupole-time-of-flight mass spectrometry. Created in BioRender, Mustonen, A.-M. (2025) https://BioRender.com/p81s444

This was an exploratory case–control study approved by the Ethical Committees of the Oulu University Hospital (decision #29/2011, amendment 2/24/2014) and Kuopio University Hospital (decision #79//2013, #73/2016) in accordance with the Helsinki Declaration. All patients had attained the age of majority and provided written informed consent. The study protocol is summarized in Fig. 1. The subjects were recruited among total knee arthroplasty (TKA) and arthroscopy patients at the Oulu University Hospital and Kuopio University Hospital. The samples were collected between 2014 and 2018, and the untargeted metabolomic analysis was conducted in 2018, followed by the targeted data processing in 2024. SF was collected from knee joints during TKA for end-stage primary KOA (*n* = 2 men, 8 women) or end-stage seropositive RA (*n* = 3 men, 7 women). The control subjects (*n* = 2 men, 3 women) were patients at the Kuopio University Hospital undergoing arthroscopy for acute meniscal or cruciate ligament trauma with no evidence of KOA/RA. The IFP samples were collected from the same KOA and RA patients during TKA, and all samples were stored at –70 °C. The KOA cases presented with clinical signs of severe OA, Kellgren–Lawrence grade 3–4, and met the criteria set by the Finnish Ministry of Social Affairs and Health for arthroplasty. The RA cases met the 1987 diagnostic criteria of the American College of Rheumatology or the 2010 updated criteria of the American College of Rheumatology/European League Against Rheumatism [14]. Seropositivity was defined by elevated concentrations of either rheumatoid factor or anti-citrullinated protein antibodies, or both. All subjects were surveyed for gender, age, body mass, height, body mass index (BMI), type of invasive procedure, operative diagnosis, and medication.

### Metabolite analysis

The present study utilized data obtained from a previously published untargeted metabolomic analysis [7] in a targeted manner to specifically examine AAs and related compounds. The rationale for this was an AA isomer, o-tyrosine (2-amino-3-(2-hydroxyphenyl)propanoic acid, CAS 2370–61-8), which we aimed to identify in order to examine its discriminatory power in these disease conditions. The detailed sample preparation and analytical methods were described in our previous publication [7]. The analysis was conducted with an ultra-high-performance liquid chromatograph (1290 LC system, Agilent Technologies, Waldbronn, Karlsruhe, Germany), coupled online to a high-resolution time-of-flight (TOF) mass spectrometry (6540 UHD Accurate-Mass Q-TOF, Agilent Technologies). The samples were analyzed using the hydrophilic interaction chromatography technique. The sample solution (2 µl) was injected onto a column (Acquity UPLC BEH Amide column, 2.1 × 100 mm, 1.7 μm, Waters Corporation, Milford, MA, USA), which was kept at 45 °C. Mobile phases, delivered at 600 µl/min, consisted of 50% (v/v, eluent A) and 90% (v/v, eluent B) acetonitrile, both containing 20 mM NH_4_HCO_2_ (pH 3). The following gradient profile was used: 0–2.5 min—100% eluent B; 2.5–10 min—100 → 0% eluent B; 10–10.01 min—0 → 100% eluent B; and 10.01–12.5 min—100% eluent B.

Mass spectrometry was equipped with a heated electrospray ionization (ESI), while both the positive and negative ionization modes were used to acquire data. The following ESI source settings were utilized: drying gas temperature 325 °C and a flow of 10 l/min, sheath gas temperature 350 °C and a flow of 11 l/min, nebulizer pressure 45 psi, capillary voltage 3500 V, nozzle voltage 1000 V, fragmentor voltage 100 V, and skimmer 45 V. N_2_ was used as the instrument gas. For data acquisition, an extended dynamic range mode (2 GHz) was utilized in the range from *m/z* 50 to 1600. The product ion spectrum was collected from the quality control (QC) samples with the automatic data-dependent manner. The TOF was calibrated daily and operated at high accuracy (< 2 ppm). Continuous mass axis calibration was performed by monitoring two reference ions (*m/z* 121.0509 and *m/z* 922.0098) from an infusion solution throughout the runs.

Data processing was targeted at AAs and related compounds, and the Mass Hunter Quantitative Analysis software (*v*B.09.00, build 9.0.647.0 for TOF, Agilent Technologies) was used for data processing and visualization. The identification of metabolites was based on the accurate mass and isotope information, as well as product ion spectra (MS/MS). In addition, metabolites were further identified with authentic standards by comparison of retention times in standards and samples. The confidence level of identification was established according to the reporting standards of the Metabolomics Standards Initiative [15]. Most metabolites included were at levels 1–2 of identification (LI) (Supplementary Table S1). The QC samples were used to monitor the stability and functionality of the system throughout the sample analysis (3 QCs per sample type). The analysis was semi-quantitative and based on comparison of analyte peak areas between the study groups.

### Statistical analysis

The statistical and pathway analyses were performed with the IBM SPSS *v*27 (IBM, Armonk, NY, USA) and MetaboAnalyst *v*6.0 programs (https://www.metaboanalyst.ca/), respectively. The univariate analyses included the Kruskal–Wallis analysis of variance (ANOVA) for three study groups (control, KOA, and RA SFs) and the Mann–Whitney *U* test for two study groups (KOA and RA IFPs). The sex ratios between the patient groups were compared with the Fisherʼs exact test. *P*-value < 0.05 was considered statistically significant, and the results are presented as the mean ± standard error (SE). The multivariate methods utilized for the original AA data included the supervised linear discriminant analysis (LDA), carried out with the SPSS software, and the partial least squares DA (PLS-DA), performed on the MetaboAnalyst platform. Potential biomarkers for KOA and RA were selected based on the variable importance in projection (VIP) score and the area under the curve (AUC) in the receiver operating characteristic (ROC) analysis with the MetaboAnalyst software. A post hoc power analysis, performed with the G*Power 3.1.9.7 software [16], yielded an effect size of 0.698–2.333 and a statistical power (1–*β*) of 0.338–0.998.

## Results

### Baseline characteristics

There were no significant differences in the sex ratios (Fisherʼs exact test, *p* = 0.850). The KOA and RA patients were significantly older than the control group (control, 34 ± 4; KOA, 67 ± 3; RA, 72 ± 3 years, Kruskal–Wallis ANOVA, *p* = 0.002), and the KOA patients had higher average BMIs compared to the RA group (control, 27.0 ± 2.35; KOA, 31.8 ± 1.97; RA, 24.9 ± 1.50 kg/m^2^, Kruskal–Wallis ANOVA, *p* = 0.030).

### Level of identification

The retention time of o-tyrosine did not match the values mentioned in the library, and initially, we investigated certain drugs that were found to be incorrect molecules. However, by utilizing an isomer of tyrosine as a standard, we were able to identify the peak as o-tyrosine at the LI1 level. Of the other compounds included in the study, most were identified at LI1–2, while *α*-aminobutyrate, ketoglutaric acid, and xanthurenic acid were LI3 identifications, and 10 molecules were LI4 identifications (Supplementary Table S1).

### Univariate analysis

In SF, o-tyrosine levels were highest in RA, cystine and cysteine levels were elevated in both KOA and RA compared to control, and methionine and valine levels were higher in control than in KOA or RA as shown in Supplementary Table S2. The differences between KOA and RA IFPs were numerous and are listed in order of significance: 3-hydroxyanthranilic acid, *β*-alanine, and 4-hydroxyproline were higher in RA, while methionine, methylmalonic acid, arginine, lysine, serine, histamine, valine, anserine, *α*-aminobutyrate, threonine, phenylalanine, glutamic acid, proline, p-tyrosine, taurine, histidine, 2-aminoisobutyric acid, alanine, leucine, aspartic acid, citrulline, glycine, tryptophan, asparagine, cystine, and 2-aminoadipic acid were higher in KOA than in RA IFPs.

### Supervised LDA

When only the SF samples were included in the supervised LDA, it classified 100% of the samples into their correct study group (control, KOA, or RA; Supplementary Fig. S1A). The principal contributors to the model were o-tyrosine, taurine, 2-aminoisobutyric acid, and methionine. The discriminant function 1 on the horizontal axis of Supplementary Fig. S1A explained 90.9% of the variance in the dataset and separated both control and KOA SFs from RA SFs. Control SFs were separated from KOA SFs by the discriminant function 2 on the vertical axis of Supplementary Fig. S1A, explaining 9.1% of the variance.

When the SF and IFP samples were analyzed together, the supervised LDA still classified 100% of the samples into their correct study group (control SF, KOA SF, RA SF, KOA IFP, or RA IFP; Supplementary Fig. S1B). The most important contributors to the model were o-tyrosine, isoleucine, creatinine, and tryptophan. The discriminant function 1 explained 82.8% of the variance in the dataset and separated all SF groups from IFP groups. Especially control SFs were separated from RA SFs by the discriminant function 2, explaining 14.4% of the variance.

### Pathway analysis

**Fig. 2:**
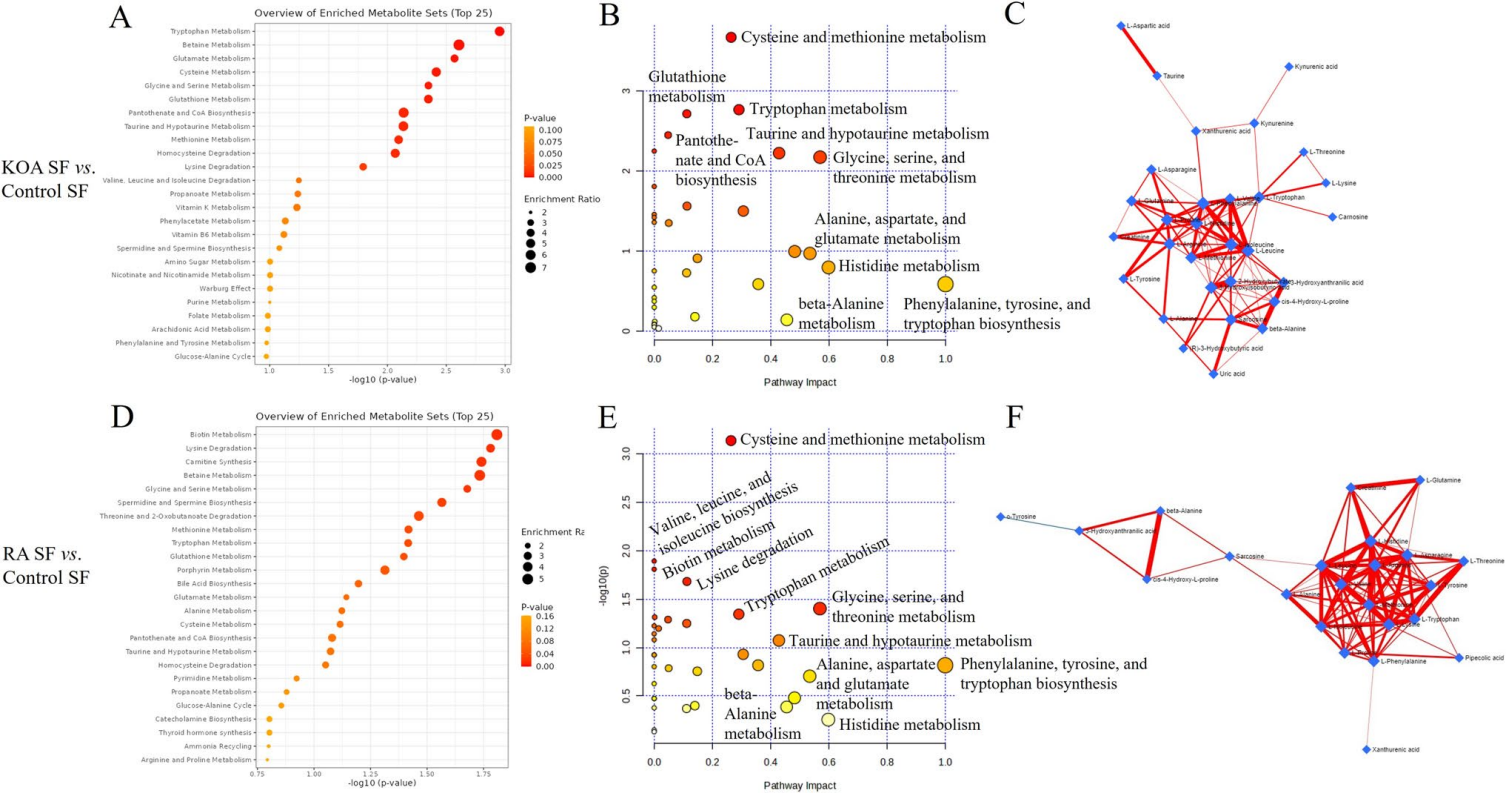
Enriched metabolite sets (**A**, **D**), key metabolic pathways (**B**, **E**), and interactive metabolic networks (**C**, **F**) in synovial fluid (SF) amino acid data from knee osteoarthritis (KOA) and rheumatoid arthritis (RA) patients compared to controls analyzed by MetaboAnalyst software

According to the metabolite set enrichment analysis of KOA and control SF samples, tryptophan metabolism, betaine metabolism, glutamate metabolism, cysteine metabolism, glycine and serine metabolism, glutathione metabolism, pantothenate and CoA biosynthesis, taurine and hypotaurine metabolism, methionine metabolism, and homocysteine degradation were the most disturbed pathways (Fig. 2A). When RA SFs were compared to control SFs, biotin metabolism, lysine degradation, carnitine synthesis, betaine metabolism, glycine and serine metabolism, spermidine and spermine biosynthesis, threonine and 2-oxobutanoate degradation, methionine metabolism, tryptophan metabolism, and glutathione metabolism emerged as the top enriched metabolite sets (Fig. 2D). To a large extent, the same pathways also had the lowest *p*-values in the pathway enrichment analysis and/or the highest impact scores in the pathway topology analysis of KOA and RA SFs *vs.* control SFs: cysteine and methionine metabolism, tryptophan metabolism, taurine and hypotaurine metabolism, glycine, serine, and threonine metabolism, phenylalanine, tyrosine, and tryptophan biosynthesis, histidine metabolism, and alanine, aspartate, and glutamate metabolism (Fig. 2B, E). Correlations of metabolic pathways in KOA and RA SFs are represented in Fig. 2C and F as interactive networks.

**Fig. 3:**
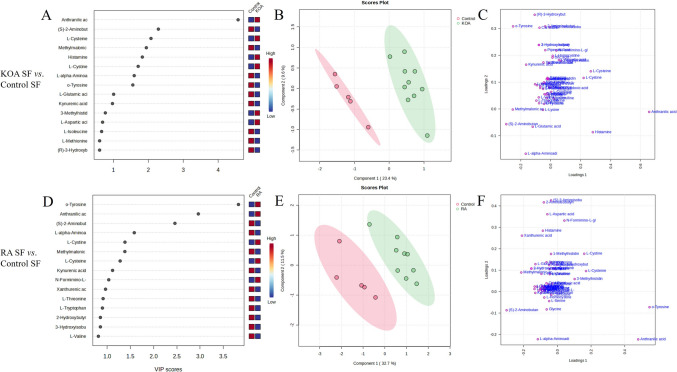
Variable importance in projection (VIP) scores (**A**, **D**), partial least squares discriminant analysis scores plots (**B**, **E**), and loadings plots of individual amino acids (**C**, **F**) in synovial fluid (SF) from knee osteoarthritis (KOA) and rheumatoid arthritis (RA) patients compared to controls analyzed by MetaboAnalyst software

The metabolites with the highest VIP scores were predominantly the same for KOA and RA SFs when compared to controls, but with different orders of importance: anthranilic acid, *α*-aminobutyrate, o-tyrosine, cysteine, methylmalonic acid, cystine, and 2-aminoadipic acid (Fig. 3A, D). Figure 3B and E show the results from the PLS-DA of SF samples, in which components 1–2 perfectly separated the control and KOA SFs, as well as the control and RA SFs, indicating perturbations in AA metabolism in both joint diseases. The AAs separating the groups in the PLS-DA were mostly the same as those with the highest VIP scores (Fig. 3C, F). The ROC analysis for KOA showed that SF cystine, cysteine, methionine, lysine, and valine had the highest AUC, specificity, and sensitivity (Table 1). For RA SF, the biomarkers showing the most potential included o-tyrosine, 4-hydroxyproline, valine, 3-hydroxyanthranilic acid, and isoleucine.

**Table 1:** Area under the curve (AUC), specificity, and sensitivity of selected amino acids for the identification of joint diseases

Comparison	Biomarker	AUC	Specificity (%)	Sensitivity (%)
KOA *vs.* control SF	Cystine	0.980	100	90
	Cysteine	0.940	100	80
	Methionine	0.920	100	80
	Lysine	0.920	100	80
	Valine	0.900	80	90
RA *vs.* control SF	o-Tyrosine	0.933	80	100
	4-Hydroxyproline	0.911	80	100
	Valine	0.911	100	80
	3-Hydroxyanthranilic acid	0.911	100	80
	Isoleucine	0.911	80	90
RA *vs.* KOA SF	o-Tyrosine	0.911	80	90
	Sarcosine	0.889	80	80
	Alanine	0.844	80	90
	Homocystine	0.833	90	90
	3-Hydroxyanthranilic acid	0.822	90	80
RA *vs.* KOA IFP	*β*-Alanine	0.940	100	80
	4-Hydroxyproline	0.940	100	80
	3-Hydroxyanthranilic acid	0.920	90	80
	Sarcosine	0.900	100	80
	Lysine	0.840	70	90

*KOA* knee osteoarthritis, *RA* rheumatoid arthritis, *SF* synovial fluid, *IFP* infrapatellar fat pad

**Fig. 4:**
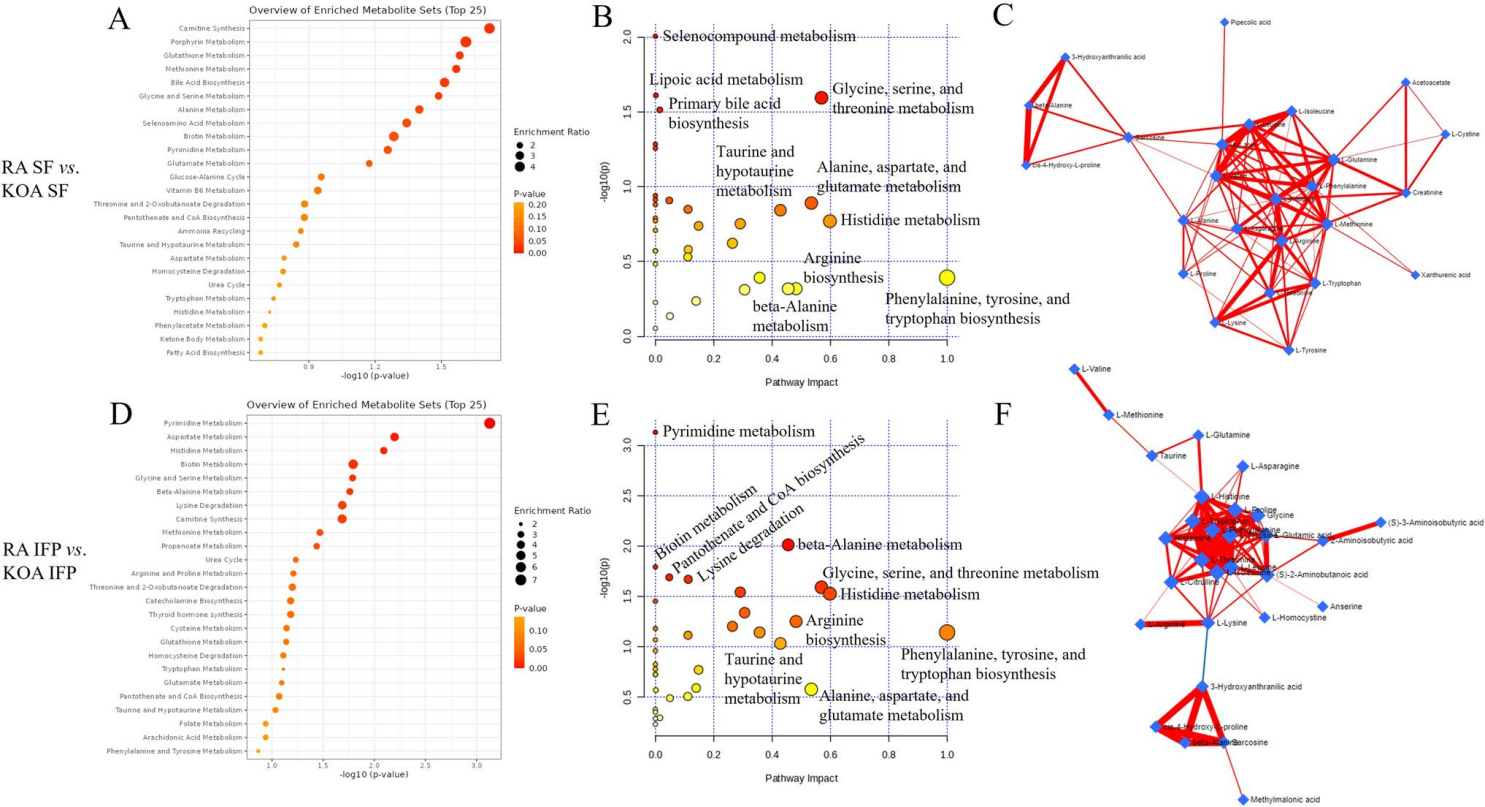
Enriched metabolite sets (**A**, **D**), key metabolic pathways (**B**, **E**), and interactive metabolic networks (**C**, **F**) in synovial fluid (SF) and infrapatellar fat pad (IFP) amino acid data from knee osteoarthritis (KOA) and rheumatoid arthritis (RA) patients analyzed by MetaboAnalyst software

**Fig. 5:**
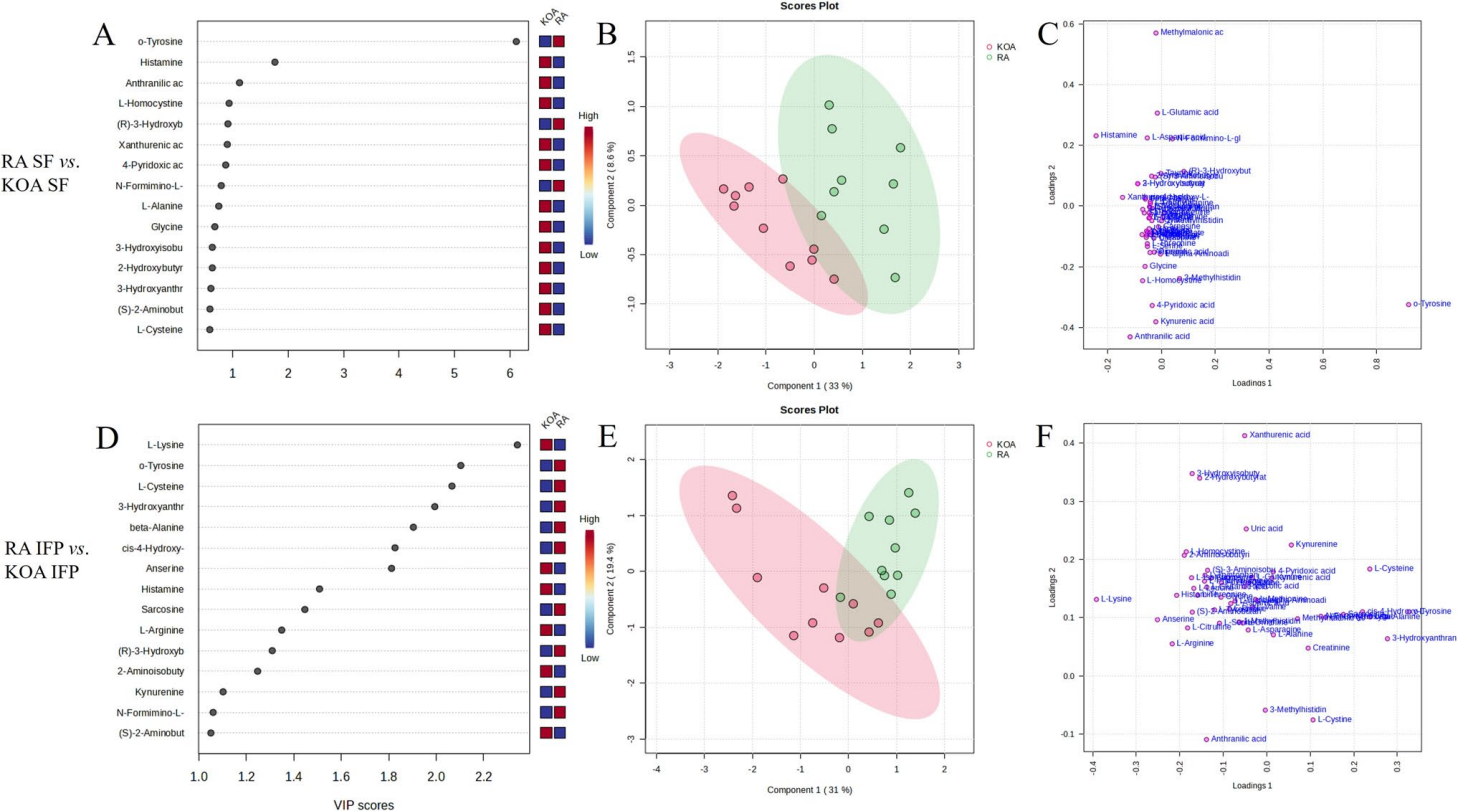
Variable importance in projection (VIP) scores (**A**, **D**), partial least squares discriminant analysis scores plots (**B**, **E**), and loadings plots of individual amino acids (**C**, **F**) in synovial fluid (SF) and infrapatellar fat pad (IFP) from knee osteoarthritis (KOA) and rheumatoid arthritis (RA) patients analyzed by MetaboAnalyst software

When KOA and RA SFs were compared to each other, the top enriched metabolite sets included carnitine synthesis, porphyrin metabolism, glutathione metabolism, methionine metabolism, bile acid biosynthesis, glycine and serine metabolism, alanine metabolism, selenoamino acid metabolism, biotin metabolism, and pyrimidine metabolism (Fig. 4A). Pathways with the lowest *p*-values and/or highest impact values included selenocompound metabolism, glycine, serine, and threonine metabolism, phenylalanine, tyrosine, and tryptophan biosynthesis, histidine metabolism, alanine, aspartate, and glutamate metabolism, and taurine and hypotaurine metabolism (Fig. 4B). Figure 4C visualizes the correlations of SF metabolic pathways in these joint diseases. The ROC analysis showed that o-tyrosine had the greatest diagnostic potential followed by sarcosine, alanine, homocystine, and 3-hydroxyanthranilic acid (Table 1). o-Tyrosine also had the highest VIP score (Fig. 5A), and PLS-DA showed a clear tendency towards the separation of KOA and RA SFs (Fig. 5B). The AAs separating the groups in the PLS-DA were mostly the same as those with the highest VIP scores (Fig. 5C).

Regarding RA and KOA IFPs, pyrimidine metabolism, aspartate metabolism, histidine metabolism, biotin metabolism, glycine and serine metabolism, *β*-alanine metabolism, lysine degradation, and carnitine synthesis emerged as pathways differentiating the diagnoses (Fig. 4D). Pathways with the lowest *p*-values and/or highest impact values included pyrimidine metabolism, *β*-alanine metabolism, phenylalanine, tyrosine, and tryptophan biosynthesis, histidine metabolism, glycine, serine, and threonine metabolism, alanine, aspartate, and glutamate metabolism, and arginine biosynthesis (Fig. 4E). Figure 4F represents the interactive networks of IFP metabolic pathways. Lysine, o-tyrosine, cysteine, 3-hydroxyanthranilic acid, *β*-alanine, 4-hydroxyproline, and anserine showed the highest VIP scores (Fig. 5D), while *β*-alanine, 4-hydroxyproline, 3-hydroxyanthranilic acid, sarcosine, and lysine had the highest AUC, specificity, and sensitivity (Table 1). PLS-DA showed some overlap between the diagnoses (Fig. 5E), and the AAs separating the groups were mostly the same as those with the highest VIP scores (Fig. 5F).

## Discussion

The present study examined alterations in intra-articular AA levels and metabolism in the SF and IFP of KOA and RA patients. Several AAs and related metabolites showed significantly different levels in controls and joint disease patients. Mostly the same metabolic pathways, such as cysteine and methionine metabolism, changed in both KOA and RA SFs compared to controls. The main difference between the diagnoses was the elevated level of o-tyrosine in RA SF that could indicate free-radical hydroxylation of phenylalanine due to oxidative stress. The metabolic pathways that differentiated KOA and RA IFPs (e.g., pyrimidine and *β*-alanine metabolisms) were partly different from those distinguishing KOA and RA SFs (e.g., selenocompound metabolism). The identified nitrogenous compounds and AA metabolism pathways could have potential as novel diagnostic and therapeutic targets for degenerative joint diseases.

RA SF samples featured disturbed phenylalanine, tyrosine, and tryptophan biosynthesis pathway and high levels of o-tyrosine, a structural isomer of p-tyrosine derived from non-enzymatic free-radical hydroxylation of phenylalanine under increased oxidative stress [17]. The ratio of o-tyrosine to p-tyrosine was also elevated in RA SF compared to control and KOA SFs (Kruskal–Wallis ANOVA, *p* = 0.005). o-Tyrosine has been previously considered a biological marker of oxidative stress, but there is accumulating evidence suggesting that it could directly contribute to the toxic effects of oxidative stress in cells and tissues via protein degradation, apoptosis, and proliferation. Oxidative stress increases with age and is likely involved in the pathogeneses of age-related diseases. This phenomenon is also known to prevail in RA that displays a rapid proliferation rate and strong invasion ability of synovial fibroblasts [18], increased local energy demands due to pannus formation, and hypoxia caused by the dysregulated microvasculature [19]. The present results suggest that the potential diagnostic value of o-tyrosine in RA warrants further investigation, ideally focusing on the routinely accessible plasma/serum samples, thereby avoiding the need for more invasive SF aspiration. Based on power calculations, a sample population of approximately 10 controls, 20 KOA patients, and 20 RA patients would yield an effect size of 0.6 and a statistical power of 0.95, representing a reasonable next step prior to clinical trials.

RA IFP was characterized by increased *β*-alanine levels that were also reflected in the observed alterations in the pyrimidine metabolism pathway [20] and, according to AUC and VIP scores, *β*-alanine emerged as a potential RA biomarker. *β*-Alanine has been previously suggested to function as a neurotransmitter, because it is an agonist of glycine and GABA_A_ receptors [20]. In addition, its plasma concentrations were found to positively associate with the duration of KOA pain [13]. As some antirheumatic drugs, such as leflunomide, are pyrimidine synthesis inhibitors [21], the current finding may also relate to the disease-modifying antirheumatic drugs used by our patients. It is plausible that, with the synthesis inhibitors, the pyrimidine metabolism pathway would show alterations in RA.

The metabolites with altered levels in KOA or RA also included sulfur-containing AAs with essential roles in protein synthesis, structure, and folding [22]. While both RA and KOA SFs showed increased levels of cysteine and cystine, suggesting perturbations in cysteine and methionine metabolism pathway in these conditions, they were still differentiated by, for instance, selenocompound metabolism. Our finding agrees with previous literature that documented elevated plasma cystine levels in KOA patients *vs.* controls [13], but it is dissimilar to a report with reduced SF cysteine levels in late-stage *vs.* early-stage KOA [23]. The plasma cystine concentrations were also found to associate with less KOA pain [13]. In KOA IFP, increased taurine, cystine, and methionine levels suggested disturbances in taurine and hypotaurine metabolism and cysteine and methionine metabolism pathways. Cysteine is critical for T cell functions, as T cells lack the enzyme that converts methionine to cysteine [24]. Therefore, the increased cystine levels could hypothetically reflect the inflammatory nature of these joint diseases. Reduced methionine levels were previously documented in OA SF [25], and we observed a positive association between plasma methionine concentrations and tibial articular cartilage thickness [13]. This essential AA is closely linked to epigenetics by providing the methyl group for histone methyltransferases [26]. According to our results, SF methionine, cysteine, and cystine show potential as biomarkers and could characterize the metabolic milieu of KOA patients.

KOA IFP also differed from RA IFP by altered phenylalanine, tyrosine, and tryptophan biosynthesis pathway reflected in the increased levels of phenylalanine and p-tyrosine, which may be associated with dopamine and catecholamine synthesis [26]. In addition, the elevated levels of tryptophan along with lower 3-hydroxyanthranilic acid—an intermediate in the metabolism of tryptophan through the kynurenine pathway [27]—could be related to serotonin synthesis [26]. However, the relevance of these neurotransmitter/hormone synthesis pathways in intra-articular fat remains obscure. 3-Hydroxyanthranilic acid had high VIP scores when comparing RA IFP to KOA IFP, and it may participate in the regulation of inflammation and oxidative stress [27]. Downregulation of tryptophan-related metabolomic profile has been previously documented in RA SF *vs.* OA SF [28]. Moreover, Kim et al. found increased SF phenylalanine levels in late-stage *vs.* early KOA [23], and we observed higher plasma phenylalanine concentrations to associate with lower pain sensitivity [13].

The glycine, serine, and threonine metabolism pathway also differed between KOA and RA IFPs, and the serine, threonine, and glycine levels were elevated in KOA. The activation and differentiation of T cells are known to require serine [24], the plasma levels of which previously showed an inverse correlation with visual analog scale global assessment of well-being in RA patients [29]. Elevated SF threonine levels were documented in OA [30], and its increased plasma concentrations associated with lower pain sensitivity [13]. Besides high glycine levels, upregulated collagen metabolism in KOA IFP may be indicated by the elevated levels of proline, alanine, arginine, and glutamic acid, among other AAs present in collagen [31].

The current study observed increased levels of several AAs linked to the urea cycle, i.e., citrulline, aspartic acid, and arginine [26] in KOA *vs.* RA IFPs. Previous studies have reported reduced plasma arginine concentrations in OA, which could reflect its increased catabolism to repair the damaged cartilage by producing more ornithine, proline, and polyamines [32]. As arginine is the nitrogenous substrate for nitric oxide synthases [26], its depletion could theoretically have beneficial effects on synovial inflammation, cartilage degradation, and pain [33]. The causes for the elevated levels of arginine in the KOA intra-articular fat of the present study remain unknown. Increased levels of proline were also observed, which could be linked to arginine metabolism or to collagen deposition due to fibrosis that occurs in KOA IFP [34].

KOA IFP also differed from RA IFP in terms of alanine, aspartate, and glutamate metabolism pathway, which has close connections to the TCA cycle [26]. This was indicated by the elevated levels of glutamic acid, aspartic acid, asparagine, alanine, and proline in intra-articular fat. Previously, increased serum alanine and 4-hydroxyproline were identified as important biomarkers distinguishing KOA patients from healthy controls [35], while we observed lower 4-hydroxyproline levels in KOA *vs.* RA IFP. Glutamic and aspartic acids are excitatory AA neurotransmitters in the central nervous system [9], and the reason for their increased levels in KOA IFP remains unresolved but could be related, e.g., to the sympathetic and sensory innervation of adipose tissue. SF asparagine was previously observed to increase from early to late-stage KOA [23], while its plasma concentrations were inversely associated with pain sensitivity [13]. Aspartic acid concentrations, on the other hand, were linked to higher pain symptoms, and those of alanine to thicker femoral articular cartilage.

BCAAs leucine and valine showed increased levels in KOA IFP compared to RA IFP. In contrast to adipose tissues, SF valine was lower in KOA and RA compared to control, and hypothetical explanations could be, e.g., reduced proteolysis or increased BCAA catabolism in these conditions. Our previous study observed positive associations of plasma BCAAs to cartilage retention and physical performance in KOA patients [13]. Serum BCAA/histidine ratio has been proposed as a useful marker of KOA severity [12]. While we found increased levels of BCAAs, histidine, histamine, and anserine in KOA IFP, the calculated BCAA/histidine ratios did not reach statistical significance. On the other hand, isoleucine/histidine and valine/histidine ratios were significantly reduced in KOA SF compared to control SF disagreeing with the previous serum data [12]. The elevated histamine level in KOA IFP was reflected in altered histidine metabolism pathway and may relate to higher inflammatory responses in our KOA patients, who had not used immunosuppressive medication, unlike the RA patients.

There are some study limitations to be considered. The data were collected using an untargeted method, which is considered semi-quantitative and provides information on differences between groups (areas) but not exact concentrations. The group size was relatively small, and the IFP data lacked control samples, because the sampling for intra-articular adipose tissue in controls would have been invasive and unethical. Due to this, IFP samples could only be harvested during TKA from KOA and RA patients, who were assigned to a surgical procedure, during which there would have been removal of intra-articular tissues regardless of the study. It is also necessary to mention that TKA patients are usually older than trauma patients and, thus, there was an inevitable age gap between study groups that could have affected the measured parameters.

To conclude, the present study examined alterations in local AA metabolism in the SF and IFP of end-stage KOA and RA patients. For SF, the significant differences included elevated levels of o-tyrosine in RA and specific sulfur-containing AAs that were altered in both joint diseases. Regarding intra-articular fat, increased *β*-alanine and 4-hydroxyproline were potential biomarkers for RA, while KOA showed elevations in AAs related to the urea cycle and arginine biosynthesis, alanine, aspartate, and glutamate metabolism, phenylalanine, tyrosine, and tryptophan biosynthesis, cysteine metabolism, and histidine metabolism. In the future, there is a need for the key markers, such as o-tyrosine, to be determined by the targeted measurement method, which can be further refined for use in clinical laboratory tests for actual patient diagnostics. A routine measurement of o-tyrosine could be especially useful as an indicator of RA.

## Supplementary Information

Below is the link to the electronic supplementary material.

Supplementary file1 (JPG 150 KB)

Supplementary file2 (PDF 607 KB)

Supplementary file3 (PDF 296 KB)

## Data Availability

All relevant data generated or analyzed during this study are included in this published article and its supplements.

## Abbreviations

*AA*: Amino acid
*ANOVA*: Analysis of variance
*AUC*: Area under the curve
*BCAA*: Branched-chain amino acid
*BMI*: Body mass index
*ESI*: Electrospray ionization
*IFP*: Infrapatellar fat pad
*KOA*: Knee osteoarthritis
*LDA*: Linear discriminant analysis
*LI*: Level of identification
*OA*: Osteoarthritis
*PLS-DA*: Partial least squares discriminant analysis
*QC*: Quality control
*RA*: Rheumatoid arthritis
*ROC*: Receiver operating characteristic
*SE*: Standard error
*SF*: Synovial fluid
*TKA*: Total knee arthroplasty
*TOF*: Time-of-flight
*VIP*: Variable importance in projection
