# Supplementary figures and images for: Synovial fluid o-tyrosine is a potential biomarker for autoimmune-driven rheumatoid arthritis
Source: Clin Rheumatol. 2025 May 31;44(7):2657–68. doi: 10.1007/s10067-025-07491-z (PMC12234615; doi:10.1007/s10067-025-07491-z)

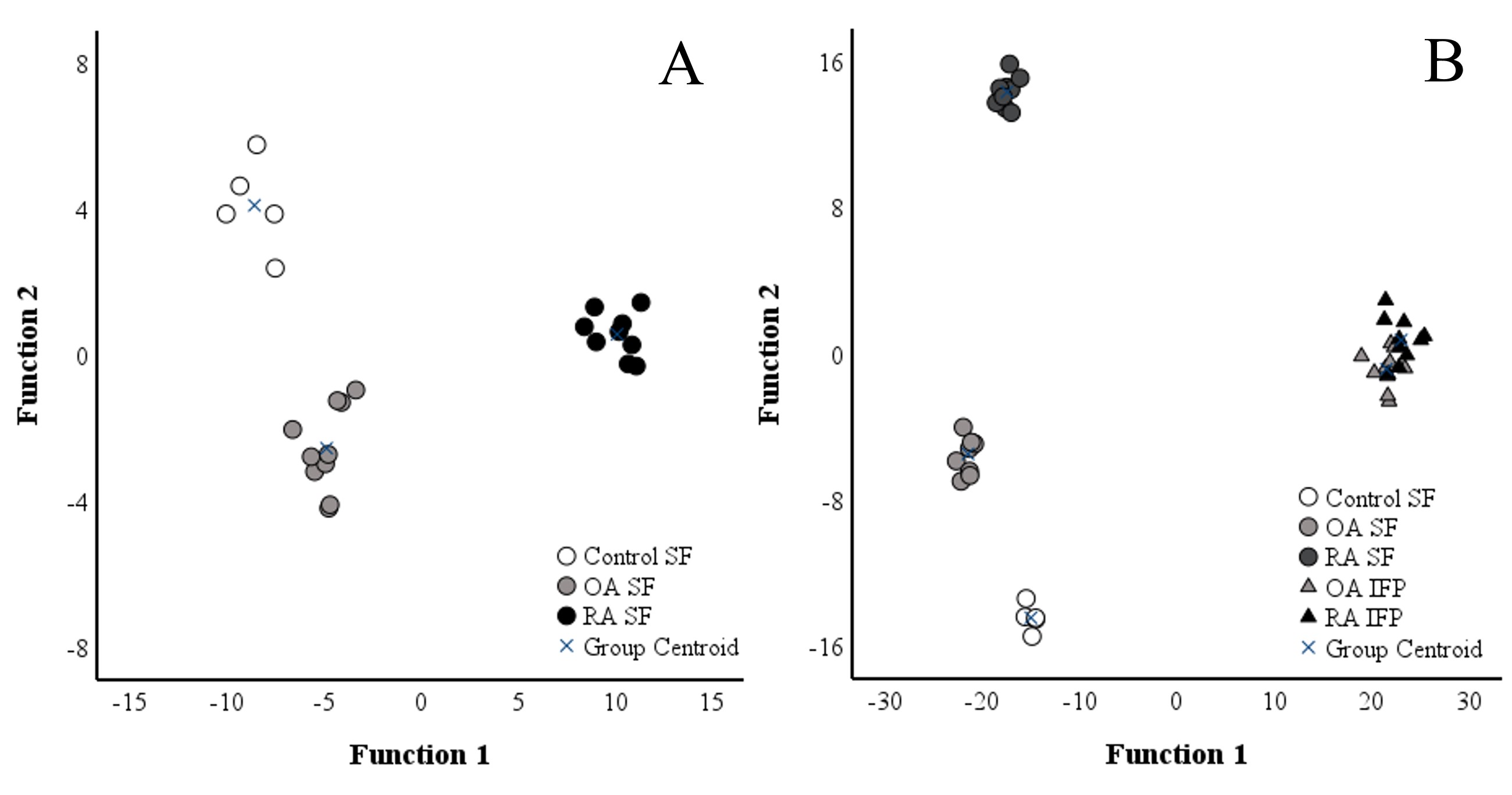

Supplement: Supplementary file 1 — Supplementary file1 (JPG 150 KB) [file 10067_2025_7491_MOESM1_ESM.jpg]
