# Supplementary material for: Synovial fluid o-tyrosine is a potential biomarker for autoimmune-driven rheumatoid arthritis
Source: Clin Rheumatol. 2025 May 31;44(7):2657–68. doi: 10.1007/s10067-025-07491-z (PMC12234615; doi:10.1007/s10067-025-07491-z)
Supplement: Supplementary file 2 — Supplementary file2 (PDF 607 KB) [file 10067_2025_7491_MOESM2_ESM.pdf]

Supplementary Table S1. Identified compounds with their metabolome database codes, levels of identification, retention times (RT), and respective peak areas (arbitrary unit, mean ± SE).

Name	HMDB ID	Level of identification	Ion Polarity	RT (min)	*m/z*	Control SF	OA SF	RA SF	OA IFP	RA IFP
1-Methylhistidine	HMDB00001	LI 1	positive	6.21	170.0924	1149458 ± 286378	1128200 ± 95224	1614834 ± 430886	442303 ± 125412	187418 ± 37951
2-Aminoadipic acid	HMDB00510	LI 1	positive	6.20	162.0761	34671 ± 20608	9546 ± 1173	14436 ± 5801	8179 ± 2354	4002 ± 840
2-Aminoisobutyrate	HMDB00112	LI 1	positive	4.52	104.0706	1558 ± 560	1591 ± 292	3815 ± 2528	3172 ± 1368	503 ± 118
3-Hydroxyanthranilic acid	HMDB0001476	LI 1	positive	5.80	154.0499	179438 ± 23473	154706 ± 10842	136742 ± 16272	96475 ± 5527	133912 ± 4578
4-Hydroxyproline	HMDB00725	LI 1	positive	5.57	132.0655	6356336 ± 591548	6091663 ± 486929	5730394 ± 1105358	5561975 ± 308817	7167208 ± 403507
Alanine	HMDB00161	LI 1	positive	5.80	90.0550	38097 ± 2006	42013 ± 2798	33896 ± 3606	30173 ± 5232	15276 ± 1883
Anserine	HMDB00194	LI 1	positive	7.04	241.1295	3431 ± 960	3542 ± 416	3390 ± 483	5561 ± 2107	998 ± 512
Anthranilic acid	HMDB0001123	LI 1	positive	5.20	138.0550	2051677 ± 1848537	5449211 ± 1015745	6080611 ± 1807361	631934 ± 191641	349772 ± 99083
Arginine	HMDB00517	LI 1	positive	6.94	175.1190	4449446 ± 328743	4322665 ± 176393	4050512 ± 216808	2062297 ± 674503	501719 ± 244008
Asparagine	HMDB00168	LI 1	positive	6.17	133.0608	247790 ± 25270	242439 ± 12039	213551 ± 14327	197775 ± 46827	83023 ± 15253
Aspartic acid	HMDB00191	LI 1	negative	6.71	134.0448	28851 ± 15128	10723 ± 1046	11098 ± 2344	148453 ± 35953	50988 ± 6621
Beta-alanine	HMDB00056	LI 1	positive	5.80	90.0550	51347 ± 3989	51237 ± 3673	49387 ± 7247	53974 ± 2317	72447 ± 3455
Carnosine	HMDB00033	LI 1	positive	7.15	227.1139	1615 ± 481	1565 ± 152	3747 ± 833	7908 ± 3359	2724 ± 787
Citrulline	HMDB00904	LI 1	positive	6.35	176.1030	1415960 ± 230438	1638591 ± 149910	1657245 ± 210720	927933 ± 291681	235742 ± 66192
Creatinine	HMDB00562	LI 1	positive	1.22	114.0662	21784576 ± 2134338	19867476 ± 1066905	21504901 ± 1107371	4240289 ± 615908	3166122 ± 410596
Cysteine	HMDB0000574	LI 1	negative	5.28	122.0270	1493 ± 422	3705 ± 228	3718 ± 472	303 ± 66	244 ± 59
Cystine	HMDB0000192	LI 1	positive	7.54	241.0311	268553 ± 57079	608294 ± 32999	702003 ± 67798	28518 ± 5586	18367 ± 5742
Glutamic acid	HMDB00148	LI 1	positive	6.50	148.0604	450306 ± 136191	219205 ± 21765	356353 ± 80863	2208325 ± 736068	498552 ± 95015
Glutamine	HMDB00641	LI 1	positive	6.30	147.0764	8404968 ± 921671	8733923 ± 245763	8751325 ± 430395	3646867 ± 982211	1635219 ± 252015
Glycine	HMDB00123	LI 1	positive	6.10	76.0393	10390 ± 2406	9710 ± 937	7835 ± 721	10079 ± 3043	3127 ± 719
Histamine	HMDB00870	LI 1	positive	5.48	112.0869	9912 ± 5675	25461 ± 9243	16825 ± 10864	4496461 ± 1533508	750045 ± 210681
Histidine	HMDB00177	LI 1	positive	7.00	156.0768	5497191 ± 446959	5171313 ± 158111	4685276 ± 207851	2835599 ± 777504	955138 ± 144874
Homocystine	HMDB00575	LI 1	negative	7.24	269.0624	2901 ± 776	3454 ± 471	2721 ± 750	10310 ± 3803	2014 ± 396
Isoleucine	HMDB00172	LI 1	positive	4.59	132.1019	3447393 ± 366575	2461955 ± 143722	2597385 ± 231010	799302 ± 299182	178429 ± 43325
Kynurenine	HMDB00684	LI 1	positive	4.37	209.0921	78729 ± 22642	65863 ± 4870	90359 ± 8727	11391 ± 3417	7528 ± 1064
Leucine	HMDB00687	LI 1	positive	4.34	132.1019	6776679 ± 566896	5409471 ± 278451	5443193 ± 402930	3394777 ± 1221360	736246 ± 161473
Lysine	HMDB00182	LI 1	positive	7.30	147.1128	3743124 ± 412274	3228007 ± 227589	2984372 ± 220951	811573 ± 348794	85187 ± 53855
Methionine	HMDB0000696	LI 1	positive	4.90	150.0583	116407 ± 7864	86218 ± 3546	92094 ± 6386	226710 ± 60071	81617 ± 7414
Phenylalanine	HMDB00159	LI 1	positive	4.30	166.0863	4343689 ± 532864	3673991 ± 147687	4081873 ± 465020	2152968 ± 750546	469180 ± 89658
Proline	HMDB00162	LI 1	positive	5.10	116.0706	5611913 ± 703131	4617828 ± 311065	4850534 ± 575916	4742485 ± 1298773	1480975 ± 261777
Sarcosine	HMDB00271	LI 1	positive	5.53	90.0550	89741 ± 6309	92869 ± 3688	83915 ± 10229	84460 ± 6026	88724 ± 5053
Serine	HMDB0000187	LI 1	positive	6.40	106.0499	1247 ± 285	1101 ± 114	1014 ± 84	3183 ± 986	854 ± 133
Taurine	HMDB00251	LI 1	positive	5.26	126.0219	43107 ± 10478	43195 ± 2891	62532 ± 10567	266796 ± 76036	93509 ± 6586
Threonine	HMDB00167	LI 1	positive	5.98	120.0655	900485 ± 224901	604376 ± 41898	558607 ± 41183	502790 ± 177622	107779 ± 22166
Tryptophan	HMDB0030396	LI 1	positive	4.36	205.0972	1963903 ± 345099	1451395 ± 126224	1310143 ± 100788	355261 ± 115863	90969 ± 21192
o-Tyrosine	HMDB0006050	LI 1	positive	4.65	182.0812	10228 ± 6358	9985 ± 4464	170027 ± 58784	259 ± 49	466 ± 123
p-Tyrosine	HMDB00158	LI 1	positive	5.30	182.0812	222984 ± 16776	206768 ± 17556	210111 ± 18084	401571 ± 117155	114091 ± 25076
Valine	HMDB00883	LI 1	positive	5.10	118.0863	1673097 ± 136679	1259899 ± 58250	1229361 ± 86292	961479 ± 254087	376781 ± 54790
Kynurenic acid	HMDB00715	LI 2	positive	2.60	190.0499	2160 ± 762	1125 ± 238	1179 ± 349	722 ± 315	428 ± 118
Methylmalonic acid	HMDB0000202	LI 2	positive	1.60	119.0339	1865 ± 763	329 ± 62	1542 ± 561	12270 ± 1920	7492 ± 718
Ornithine	HMDB00214	LI 2	positive	7.17	133.0972	629601 ± 106093	725099 ± 92722	843798 ± 87349	43684 ± 12893	18727 ± 5877
Alpha-aminobutyrate	HMDB0000452	LI 3	negative	6.00	104.0706	6119 ± 1910	1317 ± 176	1361 ± 282	26304 ± 8627	5432 ± 1382
Ketoglutaric acid	HMDB0000208	LI 3	positive	5.30	147.0288	3621 ± 1080	3342 ± 785	4126 ± 717	1410 ± 575	3301 ± 1561
Xanthurenic acid	HMDB00881	LI 3	positive	3.30	206.0448	3865 ± 1852	2707 ± 352	2303 ± 588	1025 ± 416	392 ± 139

105.0546@1.00	HMDB0000008	LI 4	negative	1.00	105.0546	18357 ± 3128	15653 ± 1933	14345 ± 2659	2484 ± 1419	789 ± 328
170.0924@6.00	HMDB0000479	LI 4	positive	6.00	170.0924	368054 ± 90782	644406 ± 149570	1107989 ± 401097	120561 ± 39824	103775 ± 51115
184.0604@0.85	HMDB00017	LI 4	positive	0.85	184.0604	16794 ± 2960	15350 ± 1288	15235 ± 2708	43727 ± 13214	31992 ± 3709
103.0390@5.10	HMDB0000060	LI 4	positive	5.10	103.0390	4519 ± 755	4878 ± 318	5421 ± 425	1721 ± 527	503 ± 92
104.0706@5.40	HMDB0003911	LI 4	positive	5.40	104.0706	1480 ± 589	1591 ± 292	3929 ± 2580	3209 ± 1359	635 ± 115
219.1339@2.30	23400779 (PubChem CID)	LI 4	positive	2.30	219.1339	119263 ± 18631	102796 ± 9786	111610 ± 17443	46295 ± 7915	52564 ± 6666
205.1183@3.30	HMDB0240347	LI 4	positive	3.30	205.1183	1387598 ± 382991	1687852 ± 282085	1911805 ± 165121	1146908 ± 324769	651058 ± 101067
175.0713@6.00	HMDB0000854	LI 4	positive	6.00	175.0713	5285 ± 1235	7126 ± 1310	16369 ± 4214	8664 ± 1539	7999 ± 1580
130.0863@4.96	HMDB00716	LI 4	positive	4.96	130.0863	24627 ± 4336	26506 ± 3784	22805 ± 2620	18583 ± 5398	8349 ± 1964
169.0356@4.20	HMDB00289	LI 4	positive	4.20	169.0356	4431 ± 605	5441 ± 239	5438 ± 662	1484 ± 491	644 ± 115

IFP = infrapatellar fat pad, OA = osteoarthritis, RA = rheumatoid arthritis, SF = synovial fluid
