# Supplementary material for: Synovial fluid o-tyrosine is a potential biomarker for autoimmune-driven rheumatoid arthritis
Source: Clin Rheumatol. 2025 May 31;44(7):2657–68. doi: 10.1007/s10067-025-07491-z (PMC12234615; doi:10.1007/s10067-025-07491-z)
Supplement: Supplementary file 3 — Supplementary file3 (PDF 296 KB) [file 10067_2025_7491_MOESM3_ESM.pdf]

Supplementary Table S2. Kruskal–Wallis analysis of variance of the synovial fluid and Mann–Whitney U test of the infrapatellar fat pad amino acids and related compounds. Only amino acids with significant differences between study groups (p <0.05) are included.

**Synovial fluid**	*P*	Direction of change
Cystine	0.002	↑ in KOA, RA vs. C
o-Tyrosine	0.004	↑ in RA vs. C and KOA
Cysteine	0.009	↑ in KOA, RA vs. C
Methionine	0.022	↑ C vs. KOA and RA
Isoleucine/histidine	0.038	↑ C vs. KOA
Valine	0.039	↑ C vs. KOA and RA
Valine/histidine	0.043	↑ C vs. KOA
**Infrapatellar fat pad**	*P*	Direction of change
3-Hydroxyanthranilic acid	0.00038	↑ in RA vs. KOA
Methionine	0.00088	↑ in KOA vs. RA
β-Alanine	0.002	↑ in RA vs. KOA
Methylmalonic acid	0.005	↑ in KOA vs. RA
Arginine	0.007	↑ in KOA vs. RA
Lysine	0.008	↑ in KOA vs. RA
Serine	0.008	↑ in KOA vs. RA
4-Hydroxyproline	0.008	↑ in RA vs. KOA
Histamine	0.008	↑ in KOA vs. RA
Valine	0.008	↑ in KOA vs. RA
Anserine	0.010	↑ in KOA vs. RA
α-aminobutyrate	0.010	↑ in KOA vs. RA
Threonine	0.010	↑ in KOA vs. RA
Phenylalanine	0.010	↑ in KOA vs. RA
Glutamic acid	0.013	↑ in KOA vs. RA
Proline	0.013	↑ in KOA vs. RA
p-Tyrosine	0.016	↑ in KOA vs. RA
Taurine	0.016	↑ in KOA vs. RA
Histidine	0.019	↑ in KOA vs. RA
2-Aminoisobutyrate	0.019	↑ in KOA vs. RA
Alanine	0.023	↑ in KOA vs. RA
Leucine	0.023	↑ in KOA vs. RA
Branched-chain amino acids	0.023	↑ in KOA vs. RA
Aspartic acid	0.028	↑ in KOA vs. RA
Citrulline	0.028	↑ in KOA vs. RA
Glycine	0.028	↑ in KOA vs. RA
Tryptophan	0.028	↑ in KOA vs. RA
Asparagine	0.034	↑ in KOA vs. RA
Cystine	0.041	↑ in KOA vs. RA
2-Aminoadipic acid	0.041	↑ in KOA vs. RA

↑ = increase in chromatographic peak area, KOA = knee osteoarthritis, RA = rheumatoid arthritis, C = control
